# Preparation of Organic–Inorganic Hybrid (Sr, Ca)CO_3_ Capsules Based on Thermoresponsive Degradable Coacervation

**DOI:** 10.1021/acsabm.5c00954

**Published:** 2025-07-24

**Authors:** Syuuhei Komatsu, Yuya Mizuno, Akihiko Kikuchi

**Affiliations:** Department of Materials Science and Technology, 26413 Tokyo University of Science 6-3-1 Niijuku, Katsushika, Tokyo 125-8585, Japan

**Keywords:** thermoresponsive polymer, liquid−liquid phase separation, Pickering emulsion, strontium ion, bone regeneration

## Abstract

Recent advances in bone regeneration materials have focused on the development of artificial bone scaffolds incorporating bioactive ions, such as strontium ions, that promote bone formation. Incorporating drug retention and release capabilities into these materials is expected to not only improve bone regeneration efficiency but also provide additional drug-derived benefits. The aim of this study is to synthesize organic–inorganic hybrid capsules with a shell containing strontium salts that can retain and release therapeutic agents. The synthesized temperature-responsive polymer formed coacervate droplets that could encapsulate hydrophobic model drugs at temperatures above the lower critical solution concentration (LCST). After preparing Pickering emulsions by mixing calcium carbonate powder and coacervate droplets in an aqueous solution, the calcium carbonate on the surface was allowed to grow crystals under various solvent conditions to produce (Ca,Sr)­CO_3_ capsules. The (Ca,Sr)­CO_3_ capsules released Sr^2+^ ions from the shell phase and also released the encapsulated hydrophobic drug from the inner coacervate phase. In vitro studies using MC3T3-E1 cells showed that exposure to these capsules increased the expression of osteogenic markers, such as alkaline phosphatase (ALP), osteopontin (OPN), and osteocalcin (OCN). Combining bone regenrative activity with controlled drug loading and release capabilities, the prepared biomaterials have potential as multifunctional scaffolds for bone regeneration strategies.

## Graphical abstract

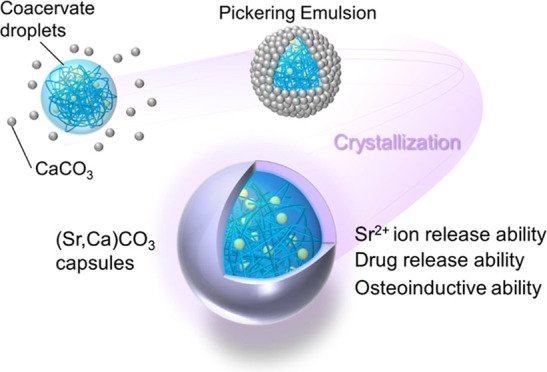

## Introduction

Bone diseases, including osteoporosis, are a global health challenge characterized by chronic pain, skeletal deformity, and increased fracture susceptibility, and treatment should be a priority in an aging society.^1^ Autologous bone grafting as a treatment for bone defects remains the clinical gold standard due to its inherent osteoconductivity, osteoinductivity, and biocompatibility, but significant limitations remain unsolved.^2,3^ Harvesting procedures carry risks such as donor site morbidity, chronic pain syndromes, and susceptibility in infection, and limited procurement limits its application for reconstruction of large defects.^4^ Artificial bones such as hydroxyapatite (HAp) and carbonate apatite have therefore attracted attention, and functional artificial bones have been reported.^5,6^ These biomaterials mimic the mineral composition of natural bone, exhibit excellent biocompatibility and customizable shapes, and promote osteoblast infiltration. Additionally, ion-substitution strategies have also been focused on, whereby bioactive metal ions (e.g., Sr^2+^, Mg^2+^, Zn^2+^) are strategically incorporated to enhance bone formation potential via activating mechanotransduction pathways and inhibiting osteoclast activity.^7–10^ Strontium, a trace element in living body, exerts dose-dependent osteogenic effects through ion modulation of cell signaling pathways. At physiological concentrations, Sr^2+^ promotes osteoblast formation by upregulating osteogenesis-related gene expression in mesenchymal stem cells via activation of Wnt/β-catenin and MAPK signaling cascades.^11,12^ However, Sr^2+^ at concentrations above physiological levels disrupts calcium homeostasis and causes strontium rickets through inhibition of new bone formation and impaired bone mineralization.^13^ To resolve this therapeutic contradiction, it is necessary to precisely control the release rate of Sr^2+^ in bone graft materials and adjust the local concentration.

Although bone regeneration capability by bioactive ion release from artificial bone is important, additional functionality can be expected by releasing drugs at the same time. For this reason, organic–inorganic hybrid materials have been attracting attention. Typically, polymer hydrogels capable of loading and releasing drugs have been reported to encapsulate HAp particles or to be composited by mineralization.^14^ Organic–inorganic hybrid artificial bones have shown good bone regeneration results.^14–16^ To enable minimally invasive implantation of artificial bones, injectable granular materials are desirable. On the other hand, designing a granular material with a well-defined structure capable of encapsulating drugs is challenging. Currently, many reports describe the introduction of drugs by adsorbing them onto the material’s surface. The drugs are released as the material surface dissolves, but this release is limited to the initial stage until the surface to which the drug is adsorbed dissolves. Therefore, a mechanism for sustained drug release or drug release responsive to environmental conditions is required.

In our previous work, we reported the fabrication of organic–inorganic hybrid capsules capable of encapsulating drugs.^17^ The capsules consist of a core of coacervate droplets containing hydrophobic drugs and a shell of calcium carbonate (CaCO_3_). The core is made of a degradable thermoresponsive polymer that undergoes phase separation above the lower critical solution temperature (LCST).^18^ A Pickering emulsion is generated by stirring the coacervate droplets formed by heating above the LCST in the presence of calcium carbonate. However, at temperatures below the LCST, the coacervate droplets dissolve and the emulsion becomes unstable. To solve this problem, we further crystallized CaCO_3_ on the droplet surface to produce stable organic–inorganic hybrid capsules. The polymer of the capsule core is degradable, and it is expected that the drug will be released in response to pH changes, such as those occurring during bone remodeling. However, the prepared capsules lacked osteogenic properties and a detailed drug release profile necessary for effective bone regeneration applications. Therefore, it is essential to design materials for core–shell hybrid capsules with a shell that has a well-defined structure capable of loading and releasing drugs while exhibiting osteogenic properties.

This study aimed to fabricate (Sr,Ca)­CO_3_ capsules, an organic–inorganic hybrid material with adjustable strontium content and drug encapsulation capacity (Figure 1). By varying the Sr^2+^ and Ca^2+^ ion concentrations in the capsule preparation conditions, (Sr,Ca)­CO_3_ capsules with different shell compositions were fabricated. By controlling the amount of SrCO_3_ introduced into the (Sr,Ca)­CO_3_ capsules, the amount of Sr^2+^ ions released from the shell could be varied. Furthermore, degradable coacervate droplets in the capsule core could potentially be utilized for drug encapsulation and release. In an in vitro study using MC3T3-E1 preosteoblasts, increased expression of osteogenic markers such as alkaline bone phosphatase (ALP), pontin (OPN), and osteocalcin (OCN) correlated with increased SrCO_3_ content in the capsules.

**Figure 1:**
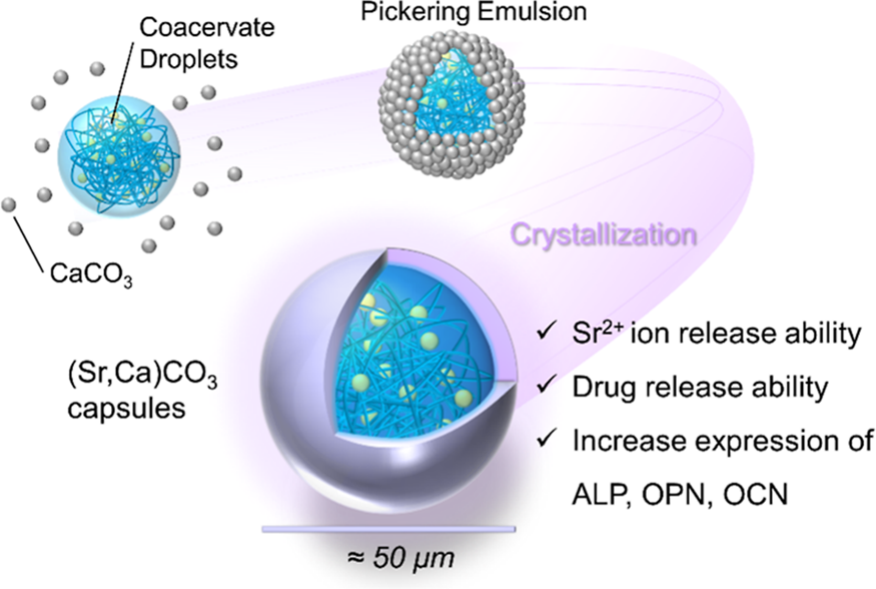
Schematic illustration of preparation of (Sr,Ca)­CO_3_ capsules.

## Experimental Section

### Materials

The following chemicals were obtained from FUJIFILM Wako Pure Chemical Corporation (Osaka, Japan): calcium carbonate (CaCO_3_), sulfuric acid (H_2_SO_4_), tetrahydrofuran (THF), rhodamine B, strontium chloride (SrCl_2_), 2,2′-azobis­(4-methoxy-2,4-dimethylvaleronitrile) (V-70), ammonium carbonate ((NH_4_)_2_CO_3_), 1,4-butanediol, dimethyl sulfoxide (DMSO), calcium chloride (CaCl_2_), 2-hydroxyethyl acrylate (HEA), Dowex 50 (H+), potassium *t*-butoxide (*t*-BuOK), 0.5% (w/v) trypsin-5.3 mmol/L EDTA•4Na solution (10-fold concentration), penicillin–streptomycin solution, labAssay ALP and Dulbecco’s phosphate buffered saline (DPBS) powder. Fetal bovine serum (FBS) was acquired from Funakoshi Co. (Tokyo, Japan). Eagle’s minimum essential medium (Eagle’s MEM Alpha Modification (MEM-α)), chloroacetaldehyde dimethylacetal were purchased from Sigma-Aldrich (MO, USA). LIVE/DEAD Viability/cytotoxicity kit was purchased from Thermo Fisher Scientific (Waltham, MA, USA). Mouse Osteopontin Assay Kit—IBL and Mouse Gla-Osteocalcin High Sensitive EIA Kit were purchased from Cosmo Bio Co. LTD (Tokyo, Japan). Prior to use, DMSO was distilled under reduced pressure conditions (95.0 °C, 0.5 kPa). MDO was synthesized via two-step reaction process following previously described methods.^19,20^

### Synthesis of Liquid–Liquid Phase Separation Type Thermoresponsive Polymer

The synthesis of thermoresponsive polymer was carried out via radical copolymerization of MDO and HEA, utilizing V-70 as the radical initiator (2.0 mol % relative to total monomer concentration) in DMSO under a nitrogen atmosphere (Scheme 1). The reaction proceeded at 30 °C for 24 h, following a previously reported protocol.^18^ The resulting polymer solution underwent dialysis against methanol for 3 days, followed by ultrapure water for 5 days, using regenerated cellulose membrane tubing (Spectra/Por3, MWCO: 3500). The dialysis ultrapure water was replaced daily, and the final product was recovered through lyophilization. Characterization of the synthesized copolymer composition was measured using ^1^H NMR spectroscopy (AVANCE Neo 400, Bruker) using DMSO-*d*_6_. The polydispersity and number-average molecular weight were determined by size exclusion chromatography (SEC) at 45 °C, employing Tosoh columns TSK gel guard column HHR-L, TSKgel G3000HHR and TSKgel G5000HHR in series, with HPLC grade DMF containing 10 mmol L^–1^ LiCl as the eluent. The thermoresponsive behavior of the prepared copolymer was evaluated by measuring temperature-dependent transmittance changes using a UV–vis spectrophotometer (V-630Bio, JASCO, Tokyo, Japan) equipped with a Peltier thermostat (ETC-717, OPS-512 TYPE, JASCO). Measurements were conducted at a wavelength of 500 nm with a heating rate of 1.0 °C/min. The temperature at which 50% transmittance was observed was defined as the LCST.

**Scheme 1:**
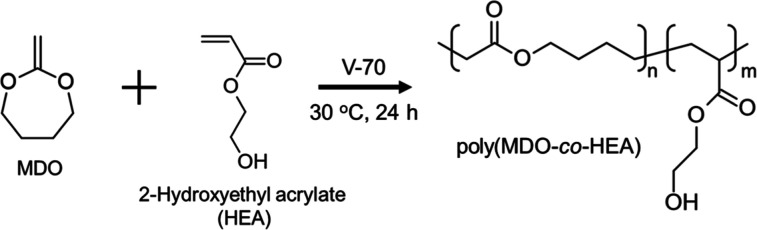
Synthesis of Liquid–Liquid Phase Separation Type Polymer, poly­(MDO-*co*-HEA)

### Preparation of Organic–Inorganic Hybrid (Sr, Ca)­CO_3_ Capsules

The preparation of (Sr, Ca)­CO_3_ capsules was conducted by modifying previously reported conditions. First, Pickering emulsions were fabricated by mixing aqueous solution containing 2.0 wt % poly­(MDO-*co*-HEA) and CaCO_3_ (10 mg mL^–1^) under continuous stirring for 2.5 h at 30 °C in 0.5 mol L^–1^ Ca^2+^ and Sr^2+^ ion mixed solution (Table 1). This temperature was selected based on the liquid–liquid phase separation behavior of poly­(MDO-*co*-HEA), which forms coacervate droplets in this condition. The size distribution of coacervate-based Pickering emulsion droplets was analyzed using a fluorescence microscope (BZ-8100, Keyence Corporation, Osaka, Japan) equipped with a temperature-controlled microwarm plate (Kitazato KM-01, Shizuoka, Japan). This setup enabled precise diameter measurements under controlled thermal conditions. Prepared droplets were deposited on glass coverslips for analysis. The average diameter was calculated from three microscopic images, with 20 randomly selected particles measured per image. For fluorescent microscopic observation (Keyence BZ-8100), rhodamine B was employed as a relatively hydrophobic fluorescent low molecular weight probe at a concentration of 1.0 μg mL^–1^. In addition, the stability of the Pickering emulsion was assessed by monitoring the temporal change in particle size.

**Table 1:** Preparation Condition of Pickering Emulsion and (Ca, Sr)­CO_3_ Capsules^a^

sample	SrCl_2_ aq/CaCl_2_ aq (volume ratio)	SrCl_2_ aq (mL)	CaCl_2_ aq (mL)
Ca-10	0:10	0	3.0
Sr-3	3:7	0.9	2.1
Sr-5	5:5	1.5	1.5
Sr-7	7:3	2.1	0.9
Sr-10	10:0	3.0	0

a Polymer conc.: 2.0 wt %, CaCO_3_: 10 mg mL^–1^.

The CaCO_3_ crystallization process on Pickering emulsion surfaces was performed under controlled CO_2_ atmosphere conditions using a sealed glass reaction vessel (8.0 × 8.0 × 8.0 cm^3^) fitted with a Teflon-sealed lid. The system incorporated 10 g of ammonium carbonate per vessel as a CO_2_ source via thermal decomposition, paired with 20 mL of concentrated sulfuric acid to chemically neutralize evolved ammonia vapors. The mineralization reaction proceeded for 6 days at 37 °C, maintaining physiological temperature conditions while ensuring gradual carbonate deposition through controlled gas-phase reagent delivery. This setup was implemented immediately following the preparation of the Pickering emulsion. The physical properties of the resultant CaCO_3_ capsules, specifically their diameter and shell thickness, were quantified using scanning electron microscopy (SEM) and SEM-energy dispersive X-ray spectroscopy (SEM, JSM-6060LA, JEOL, Tokyo, Japan). Analysis was performed on five SEM images, with two particles randomly selected per image. The average dimensions were calculated from these measurements. The surface composition of the capsules was evaluated by XRD (XRD-6100, SIMADZU, Kyoto, Japan). The freeze-dried sample (10 mg) was placed on a glass plate to prepare the measurement sample. XRD measurements were conducted using a Cu target cathode with operational parameters set at 40 kV tube voltage and 30 mA tube current. The diffraction patterns were collected over a 2θ range of 10°–50° at a scanning rate of 2.0°/min. The Sr^2+^ ion release profiles were measured by ICP-AES 60 mg of the capsule was immersed in 30 mL of ultrapure water, and the concentration was adjusted to 2 mg mL^–1^. After 1, 2, 4, and 7 h, the solution was sampled and the Sr^2+^ ion concentration was measured. A calibration curve was created using a strontium solution of known concentration, and the amount of Sr^2+^ ion released from the capsule was calculated.

### Preparation of Rhodamine B Loaded (Sr, Ca)­CO_3_ Capsules and Release Profile

Rhodamine B inclusion capacity of (Ca, Sr)­CO_3_ capsules was evaluated using the following protocol: A 20 mg sample of (Ca, Sr)­CO_3_ capsules was suspended in 5.0 mL of a 1.0 μg mL^–1^ rhodamine B aqueous solution. The suspension was then incubated at 37 °C under dark conditions for 24 h. Following incubation, the capsules underwent three times washing procedure with distilled water at 37 °C to remove any nonencapsulated rhodamine B. The purified capsules were subsequently recovered through lyophilization. Fluorescence microscopic observation (Keyence BZ-8100, Osaka) was employed to visualize the rhodamine B loaded (Ca, Sr)­CO_3_ capsules. Loading efficiency and loading amount of rhodamine B was quantitatively determined by spectrofluorometric analysis of the supernatant. Specifically, the concentration of unencapsulated rhodamine B in the supernatant was measured using a fluorescence spectrophotometer (FP-6500; JASCO, Tokyo, Japan) (λ_Ex_: 552 nm, λ_Em_: 588 nm), allowing for the calculation of the amount of rhodamine B into the capsules. The loading content and loading efficiency of rhodamine B were quantified using the following equations

1 $$loading\;content\;\left(\%\right)=\frac{weight\;of\;drug\;in\;nanoparticles}{Weight\;of\;nanoparticles}\times 100$$

2 $$loading\;efficiency\;\left(\%\right)=\frac{weight\;of\;drug\;in\;nanoparticles}{weight\;of\;feedd\;rug}\times 100$$

The release profile was evaluated under neutral conditions (pH 7.4) and in an acidic environment (pH 4.5) during bone remodeling. Rhodamine B-loaded (Ca, Sr)­CO_3_ capsules (30 mg) were suspended in 30 mL of either 0.150 mol L^–1^ acetate buffer (pH 4.5) or phosphate-buffered saline (PBS, pH 7.4) to evaluate release kinetics under different physiological conditions. The suspensions were maintained at a physiological temperature of 37 °C throughout the experiments. Aliquots of the supernatant were collected at predetermined time intervals. The concentration of released rhodamine B in these aliquots was quantified using spectrofluorometric analysis. This time-dependent measurement of rhodamine B concentration in the release medium allowed for the characterization of the release profile from the (Ca, Sr)­CO_3_ capsules under both acidic and physiological pH conditions.

### Cytotoxicity Evaluation of (Ca, Sr)­CO_3_ Capsules

The cytotoxicity of (Ca, Sr)­CO_3_ capsules was evaluated using MC3T3-E1 preosteoblast cells. For this assay, MC3T3-E1 cells were seeded in 24-well plates at a density of 1.0 × 10^4^ cells/cm^2^ and cultured for 24 h at 37 °C to allow for cell adhesion and initial proliferation. Following medium replacement, cells were treated with (Ca, Sr)­CO_3_ capsules suspended in 500 μL of MEM-α at concentrations ranging from 2.5 to 12.5 mg mL^–1^ and incubated for 24 h at 37 °C. The cells were then washed with sterile PBS and stained for 30 min using a solution containing 1.0 μmol L^–1^ calcein AM and 2.0 μmol L^–1^ ethidium homodimer III (EthD-III). Cell viability was then evaluated through fluorescence microscopy, with calcein AM indicating live cells through green fluorescence due to intracellular esterase activity, and EthD-III identifying dead cells with compromised plasma membranes through red fluorescence.

### Effect of (Ca, Sr)­CO_3_ Capsules on Differentiation of MC3T3-E1 Cells

MC3T3-E1 cells were seeded at a density of 1.0 × 10^4^ cells cm^–2^ in 2.0 mL of serum-supplemented MEM-α medium. After 24 h, the culture medium was replaced with 2.0 mL fresh medium. Subsequently, five distinct types of (Ca, Sr)­CO_3_ capsules (designated as Ca-10, Sr-3, Sr-5, Sr-7, and Sr-10) were prepared for cell exposure. For each capsule type, 15 mg sample was suspended in 500 μL of serum-containing MEM-α medium and added to the respective cell cultures. The cultures were maintained by supplementing with 500 μL of serum-containing MEM-α medium every 48 h for 14 days. To assess osteogenic differentiation, the expression levels of key markers, including alkaline phosphatase (ALP), osteopontin (OPN), and osteocalcin (OCN), were quantified using commercially available measurement kits with proposed protocols in each kit.

### Statistical Analysis

Data are expressed as the mean with standard deviation with indicated experiments or samples. The expression levels of bone differentiation markers were evaluated using the τ2 statistic. Results with p values < 0.05 were considered significant.

## Results and Discussion

### Synthesis of Thermoresponsive Polymer and Formation of Pickering Emulsion

In accordance with our previous study,^18^ thermoresponsive polymer was prepared through the radical copolymerization of MDO and HEA. These characterizations of prepared copolymers were summarized in Table 2. The synthesized polymer exhibited a molecular weight of approximately 50,000, and its composition could be modulated by adjusting the monomer feed ratio during the polymerization process. Notably, we observed a correlation between the MDO introduction and the lower critical solution temperature (LCST) of the resulting copolymer. As illustrated in Figure 2a, increasing the MDO content led to a decrease in the LCST. This phenomenon can be attributed to the enhanced hydrophobicity imparted by the MDO units, which facilitates polymer chain aggregation at lower temperatures. Subsequently, the LCST of the copolymer (MDO composition rate 9.9 mol %) under various solvent conditions as detailed in Table 1 were measured. Figure 2b showed the relationship between the LCST and the ionic composition of the solvent. The LCST decreased with increasing concentration of strontium ions in the solvent. This phenomenon can be rationalized in the context of the Hofmeister series,^21^ which predicts that strontium ions exhibit a stronger salting-out effect compared to calcium ions. The enhanced salting-out effect of strontium ions likely promotes polymer chain dehydration and aggregation at lower temperatures, resulting in the observed decrease in LCST. All subsequent experiments were performed using sample 2 (MDO 9.9 mol %) in Table 2.

**Table 2:** Characterization of Thermoresponsive poly­(MDO-*co*-HEA)

sample	MDO/HEA in feed (molar ratio)	*M*_n_ (×10^4^)^a^	*M*_w_/*M*_n_^a^	MDO composition rate in the copolymer (mol %)^b^	yield (%)	LCST (°C)^c^
1	20:80	5.2	1.6	4.1	49.3	33.4
2	30:70	5.1	1.6	9.9	59.1	21.2

a Determined by GPC. Poly­(ethylene glycol) (PEG) was used as a molecular weight standard.

b Determined by ^1^H NMR in DMSO-*d*_6_.

c Determined by UV–vis. in PBS at 500 nm (polymer conc.: 0.5 wt %).

**Figure 2:**
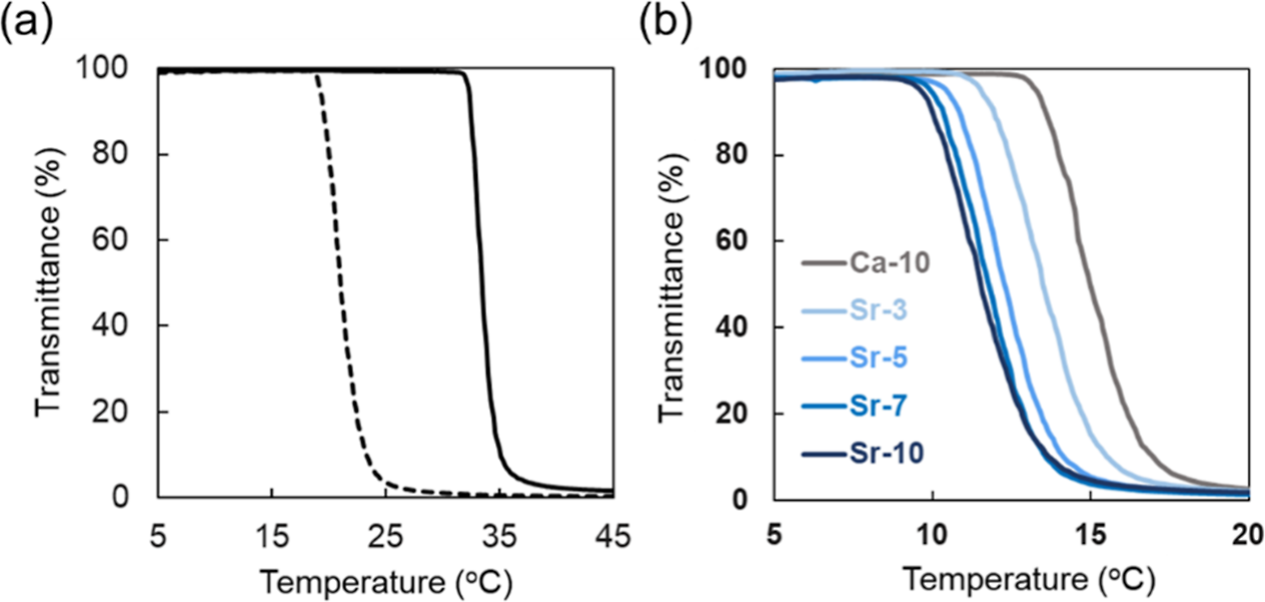
Thermoresponsive behavior of the prepared polymer in various condition. (a) Change in LCST due to differences in MDO composition in the copolymer. Solid line: MDO composition rate 4.1 mol %, dashed line: MDO composition rate 9.9 mol %. (b) Change in LCST due to differences in ion species in the solvent (the captions in the figures correspond to those in Table 1, sample 2 in Table 2.).

The phase behavior and stability of coacervate systems were systematically investigated under various conditions outlined in Table 1. The coacervate droplets consistently formed through liquid–liquid phase separation when the temperature exceeded the lower critical solution temperature (LCST) across all experimental conditions using sample 2 in Table 2 (Figure 3a). Subsequent heating of these systems in the presence of calcium carbonate particles resulted in the formation of Pickering emulsions under all tested solvent conditions in Table 1 (Figure 3b). This observation aligns with our previous study,^17^ where calcium carbonate demonstrated adsorption at the coacervate droplet interface, a phenomenon attributed to the high surface energy (Figure 3c). Time-dependent size analysis revealed distinct stability profiles. The coacervate droplets gradually increased in size and completely separated into two layers after 20 min (Figure 3 d). In stark contrast, Pickering emulsions maintained consistent dimensions throughout the observation period (Figure 3e). This improvement in stability is thought to be due to the adhesion of calcium carbonate to the surface of the coacervate droplets, which suppresses fusion between the droplets due to the presence of a shell, and causes the droplets to aggregate due to a decrease in surface energy.^17,22^ Therefore, by stirring the coacervate droplets in the presence of calcium carbonate, stable Pickering emulsions without particle size change could be easily prepared under all solvent conditions.

**Figure 3:**
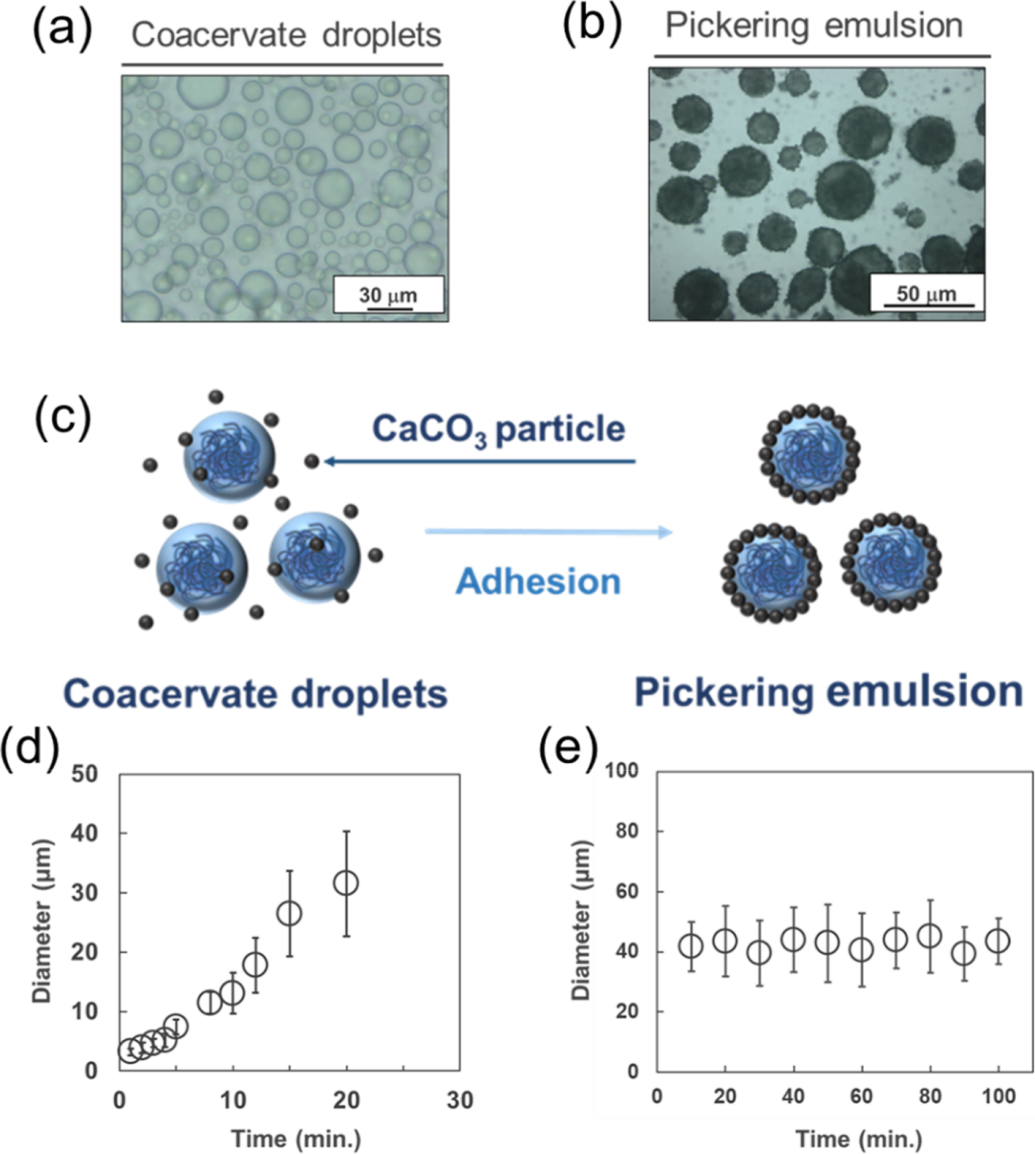
Characterization of Pickering emulsion using sample 2 in Table 2. (a) Photomicrography of coacervate droplets above LCST. (b) Photomicrography of pickering emulsions made with coacervate droplets and CaCO_3_. (c) Schematic illustration of method of pickering emulsion using coacervate droplets and CaCO_3_. (d) Time-dependent changes in the particle diameter of coacervate droplets in SrCl_2_ aq./CaCl_2_ aq. (7:3) condition. Data are expressed as the mean ± SD (*n* = 3) (e) Time-dependent particle size change of pickering emulsions in SrCl_2_ aq./CaCl_2_ aq. (7:3) condition. Data are expressed as the mean ± SD (*n* = 3).

### Preparation and Characterization of (Ca, Sr)­CO_3_ Capsules

The pickering emulsion system was subjected to a mineralization process to fabricate (Ca, Sr)­CO_3_ capsules. Following emulsion stabilization, the system was maintained under a CO_2_ atmosphere at 37 °C for 6 days to facilitate calcium carbonate crystallization on the emulsion droplet surfaces. The mineralization conditions corresponded to those detailed in Table 1, with each sample nomenclature reflecting its specific production parameters. Optical microscopy confirmed successful capsule formation across all solvent compositions (Figure 4a–e), while scanning electron microscopy (SEM) analysis revealed spherical morphology of the resulting capsules (Figure 4f). Although Sr-7 capsules are presented as a representative example in Figure 4f, analogous morphological characteristics were observed for all synthesized samples. Quantitative analysis demonstrated that the particle size of the (Ca, Sr)­CO_3_ capsules remained constant at approximately 50 μm across all experimental conditions, indicating that solvent composition did not significantly influence the final capsule dimensions. This uniformity in capsule size suggests that the mineralization process is primarily governed by the initial Pickering emulsion template rather than the specific ionic composition of the solvent medium. The crystalline phase composition of (Ca, Sr)­CO_3_ capsules was analyzed using XRD. XRD patterns revealed distinct structural characteristics across the capsule variants (Figure 4h). The Ca-10 capsules exhibited characteristic diffraction peaks at 2θ = 29.4° and 39.4°, corresponding to the (104) and (113) crystallographic planes of calcite-phase CaCO_3_. In contrast, Sr-containing capsules (Sr-3, Sr-5, Sr-7, Sr-10) demonstrated additional peaks at 25.2°, consistent with the (111) plane of strontianite-phase SrCO_3_, while retaining diminished calcite signatures. The calcium composition in the shell decreasing was observed with increasing Sr^2+^ concentration in the mineralization medium, suggesting cation exchange within the (Ca, Sr)­CO_3_ capsule shells during crystallization. Furthermore, compositional analysis of the (Ca, Sr)­CO_3_ capsules using SEM–EDX revealed a direct correlation between the Sr^2+^ concentration in the crystallization medium and the SrCO_3_ content in the capsule shells (Figures 4i and S1). The SrCO_3_ composition in the shells increased progressively with higher Sr^2+^ concentrations during crystallization, reaching up to approximately 90%. These results indicated that the crystallization dynamics of calcium carbonate and strontium carbonate under controlled crystal growth conditions and propose that (Ca, Sr)­CO_3_ hybrid capsules with tunable composition gradients were prepared. Time-dependent release profiles of Sr^2+^ ions from the (Ca, Sr)­CO_3_ capsules were observed and characterized (Figure 4j). The release amount of Sr^2+^ ion were increased with increasing the SrCO_3_ content in the shell of the (Ca, Sr)­CO_3_ capsules. This result indicated that Sr^2+^ ions can be released from the capsules shell, and the release amount can be modulated by altering the shell composition. Control of the concentration of strontium ions released into the bone environment is essential, as elevated strontium ion concentrations have been associated with adverse effects, such as inhibition of bone nodule formation and the development of osteomalacia. These effects are typically reported at concentrations of 20 μg mL^–1^ or higher.^23^ In this study, the strontium ion concentrations released from the (Ca,Sr)­CO_3_ capsules were below this value, indicating that the capsules are not releasing levels of strontium ions that could be considered cytotoxic or harmful to bone formation.

**Figure 4:**
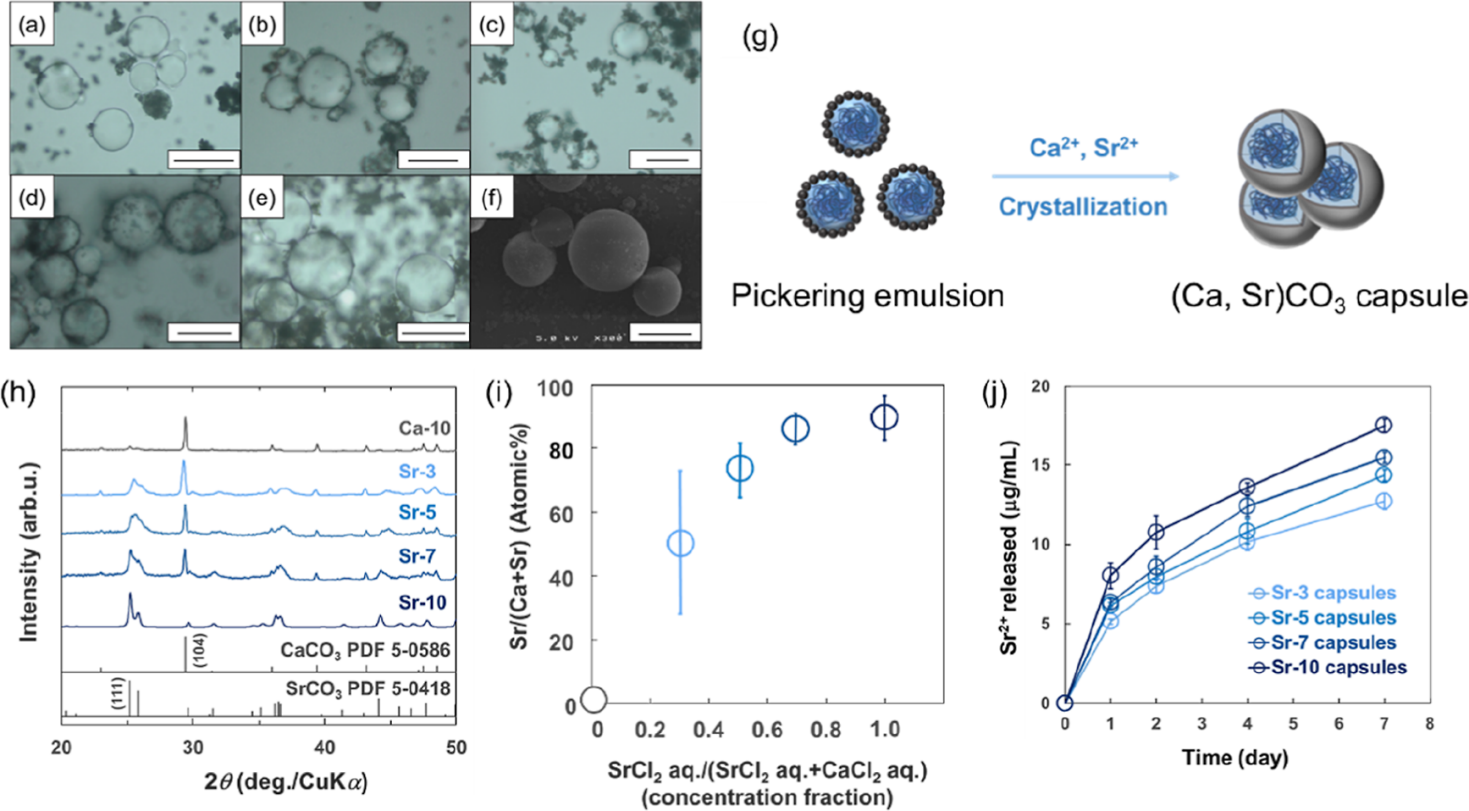
Characterization of (Ca, Sr)­CO_3_ capsules using sample 2 in Table 2. (a–e) Microscopic observation of (Ca, Sr)­CO_3_ capsules prepared under the conditions shown in Table 1. (a) Ca-10, (b) Sr-3, (c) Sr-5, (d) Sr-7, and (e) Sr-10, respectively. (f) SEM image of Sr-7 capsules. (g) Schematic illustration of (Ca, Sr)­CO_3_ capsules prepared via crystallization of pickering emulsion. (h) XRD pattern of (Ca, Sr)­CO_3_ capsules. (i) Measurement of the relationship between the amount of strontium added and the amount introduced using SEM–EDX. Data are expressed as the mean ± SD (*n* = 3) (j) Sr^2+^ ion release profile from (Ca, Sr)­CO_3_ capsules measured using ICP-AES. Data are expressed as the mean ± SD (*n* = 3).

These drug loading and release capabilities of (Ca, Sr)­CO_3_ capsules were systematically evaluated using rhodamine B as a hydrophobic model compound. Bright-field microscopy analysis confirmed the forming the Pickering emulsion at 37 °C (Figure 5a). Meanwhile, fluorescence microscopy analysis revealed clear localization of rhodamine B within the capsules (Figure 5b). This result was attributed to the concentrating properties of the coacervate droplets inside the capsules, which effectively sequester the hydrophobic molecules within the capsules. Consistent encapsulation performance was demonstrated in all the samples prepared, with an loading efficiency of 60% and a loading amount of 150 ng mg^–1^-capsules in all samples (Table 4). These results suggest that passive diffusion of hydrophobic low molecular weight model drugs through the capsule shell occurs, followed by effective retention within the coacervate droplets.

**3:** Diameter of (Ca, Sr)­CO_3_ Capsules Calculated from microscopic Observation

	Ca-10	Sr-3	Sr-5	Sr-7	Sr-10
diameter^a^ (μm)	49.0 ± 16.2	47.0 ± 12.3	48.6 ± 14.2	53.4 ± 17.6	50.0 ± 19.9

a Data are expressed as the mean with SD (*n* = 3).

**Figure 5:**
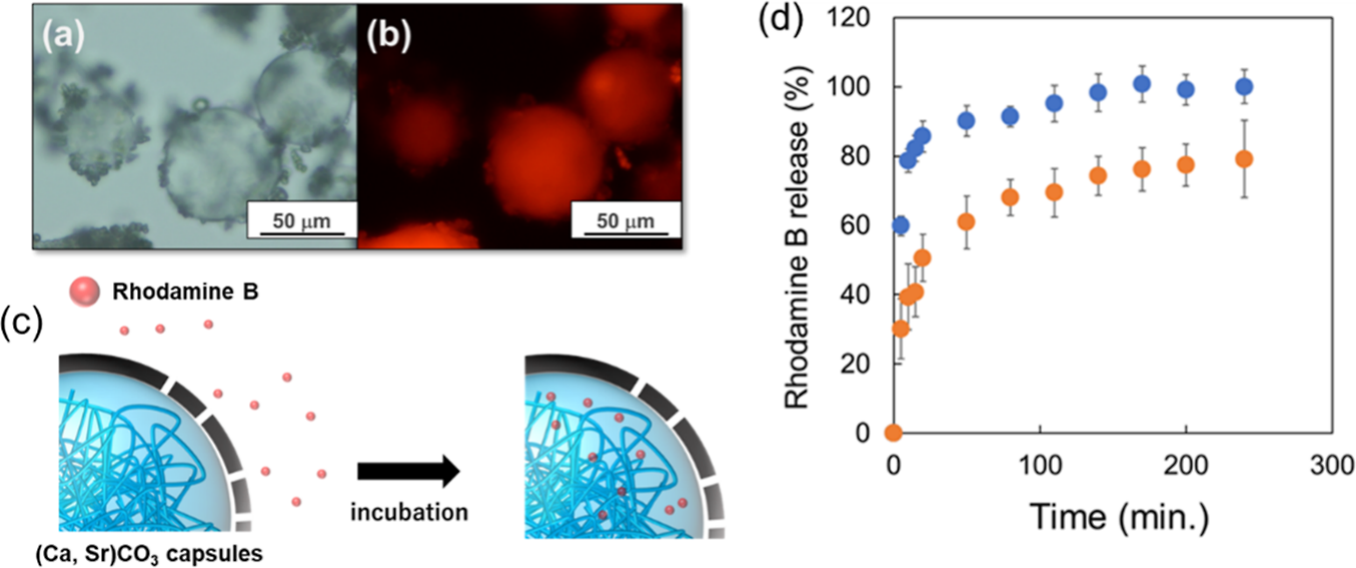
Characterization of drug loading ability and release profile of (Ca, Sr)­CO_3_ capsules. (a) Microscopic observation of (Ca, Sr)­CO_3_ capsules (Sr-7 capsules) after loaded rhodamine B. (b) Fluorescent image of rhodamine B loaded (Ca, Sr)­CO_3_ capsules (Sr-7 capsules). (c) Illustration of drug loading mechanism into (Ca, Sr)­CO_3_ capsules. (d) Rhodamine B release profile from (Ca, Sr)­CO_3_ capsules (Sr-7 capsules) in neutral (orange plot: in PBS, pH 7.4) and acidic condition (blue plot: in acetate buffer, pH 4.5). Data are expressed as the mean ± SD (*n* = 3).

**Table 4:** Loading Efficiency and Amount of Rhodamine B in (Ca, Sr)­CO_3_ Capsules

	Ca-10	Sr-3	Sr-5	Sr-7	Sr-10
loading efficiency (%)	62.9 ± 1.58	63.2 ± 9.92	59.8 ± 3.71	60.8 ± 1.63	61.1 ± 3.70
loading amount (ng mg^–1^-capsules)	157 ± 3.9	158 ± 24.8	150 ± 9.3	152 ± 4.1	153 ± 9.3

The release study of rhodamine B from (Ca, Sr)­CO_3_ capsules containing rhodamine B was carried out in pH 7.4 and pH 4.5 conditions. The latter simulated the acidic environment during bone remodeling. At pH 7.4, rhodamine B was released over time, reaching approximately 70% after 3 h. In contrast, at pH 4.5, nearly 100% release was observed within 3 h. In pH 7.4 environments, the release mechanism is mainly diffusion driven. In acidic environments, the release was promoted by hydrolysis of the polymer forming inner coacervate droplets and increased solubility of the shell. As a result, the amount of release was found to increase in acidic condition. This behavior was attributed to the change in physical properties caused by pH switching, indicating that more precise release control is needed for practical applications.

### In Vitro Cell Culture Study in the Presence of (Ca, Sr)­CO_3_ Capsules

The cytocompatibility and osteogenic differentiation potential of (Ca, Sr)­CO_3_ capsules were evaluated using MC3T3-E1 cells. Fluorescence viability assays revealed minimal cytotoxicity across all capsule formulations, with cell viability exceeding 90% at a concentration of 7.5 mg mL^–1^ (Figure 6a,b). While the Ca-10 formulation exhibited marginally reduced viability accompanied by sporadic ethidium homodimer III staining, Sr-containing capsules (Sr-3 to Sr-10) demonstrated viability metrics indistinguishable from capsule-free controls, confirming their biosafety profile. MC3T3-E1 cells were cultured under optimized conditions with 7.5 mg mL^–1^ of capsules, a concentration that maintained cell viability above 90% across all experimental groups. Under these noncytotoxic conditions, quantitative assessments of osteogenic differentiation markers ALP, OPN and OCN were performed to evaluate the bioactivity of (Ca, Sr)­CO_3_ capsules (Figure 6c–e). The temporal expression profiles of osteogenic markers revealed distinct differentiation patterns mediated by (Ca, Sr)­CO_3_ capsules. While alkaline phosphatase (ALP) activity showed no significant intergroup differences at day 7, a SrCO_3_ dose-dependent increase in ALP expression emerged by day 14 (Figure 6c). In contrast, osteopontin (OPN) exhibited significantly elevated expression levels in Sr-7 and Sr-10 groups as early as day 7, with this enhancement persisting through day 14 (Figure 6d). A parallel progression was observed for OCN, a late-stage differentiation marker, demonstrating Sr-dependent amplification of terminal osteoblast maturation (Figure 6e). These results suggest SrCO_3_ and CaCO_3_ provides baseline stimulation for early differentiation initiation, as evidenced by universal ALP elevation across all samples. However, sustained differentiation through intermediate OPN and late OCN stages requires Sr^2+^ incorporation, with differentiation-promoting efficacy effected to SrCO_3_ content in the capsules. These effects of Sr^2+^ were in good agreement with other research, and it was found that the (Ca, Sr)­CO_3_ capsules showed the differentiation-promoting ability derived from strontium, and that this ability increases with the amount of Sr salt introduced.

**Figure 6:**
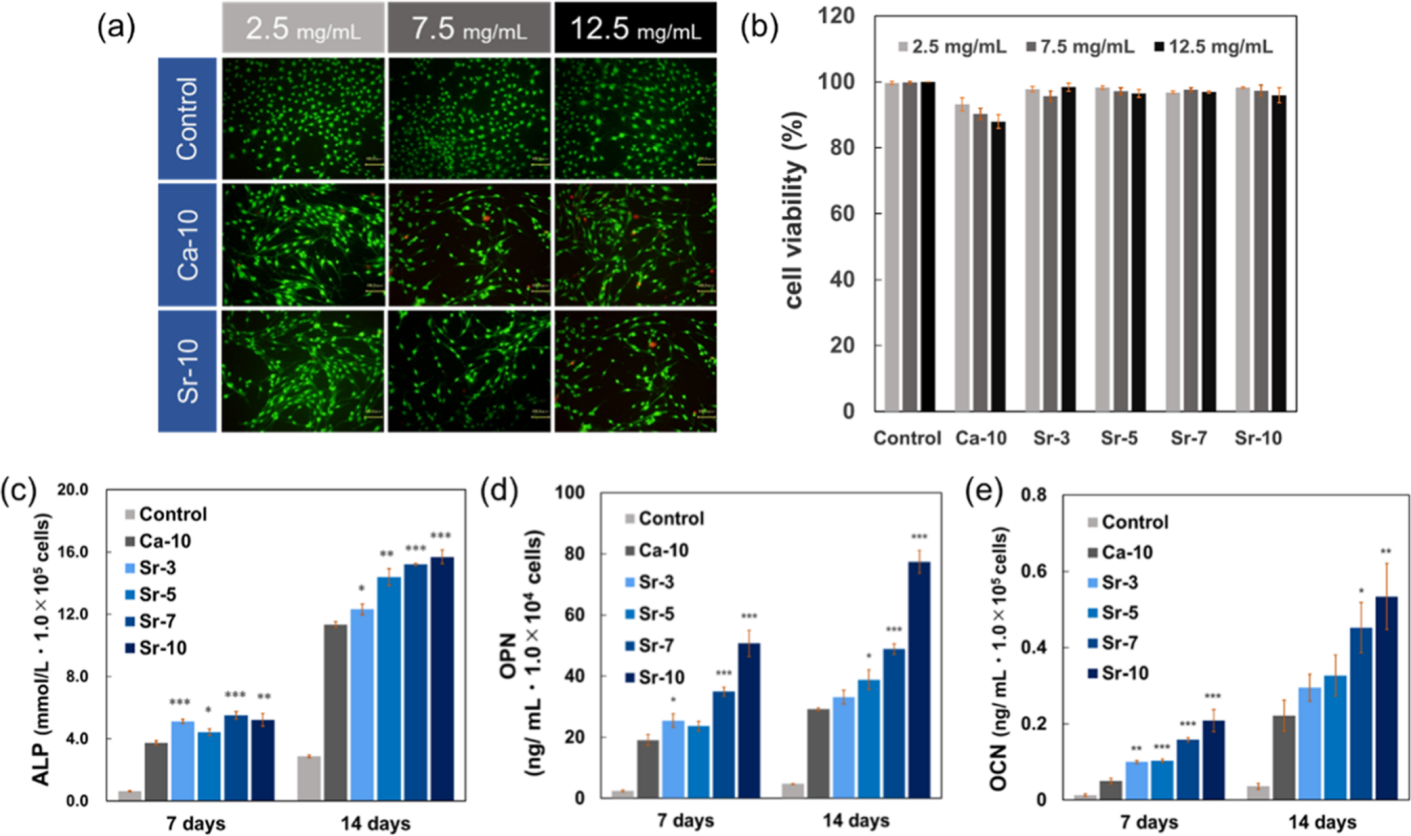
Evaluation of the effects of (Ca, Sr)­CO_3_ capsules on MC3T3-E1 cells. (a) Fluorescence images of live–dead tests of MC3T3-E1 cells with each amount of (Ca, Sr)­CO_3_ capsules (2.5, 7.5, 12.5 mg mL^–1^) after 24 h incubation. (b) Cell viability of MC3T3-E1 cells at each (Ca, Sr)­CO_3_ capsule amount (2.5, 7.5, 12.5 mg mL^–1^) after 24 h incubation. Data are expressed as the mean ± SD (*n* = 3). (c) ALP expression on the seventh and 14th days after culturing MC3T3-E1 cells in the presence of (Ca, Sr)­CO_3_ capsules (Capsules conc.: 7.5 mg mL^–1^). (d) OPN expression on the seventh and 14th days after culturing MC3T3-E1 cells in the presence of (Ca, Sr)­CO_3_ capsules (Capsules conc.: 7.5 mg mL^–1^). (e) OCN expression on the seventh and 14th days after culturing MC3T3-E1 cells in the presence of (Ca, Sr)­CO_3_ capsules (Capsules conc.: 7.5 mg mL^–1^). (c–e) Data are expressed as the mean ± SD (*n* = 3). *p < 0.05, **p < 0.01, ***p < 0.001 vs Ca-10 groups.

## Conclusion

In this study, (Sr, Ca)­CO_3_ capsules, an organic–inorganic hybrid material with adjustable strontium content and drug encapsulation capacity. The fabricated (Sr, Ca)­CO_3_ capsules allowed precise control of the amount of SrCO_3_ introduced and the regulation of the amount of Sr^2+^ ions released from the shell. Furthermore, coacervate droplets in the capsule core allowed drug encapsulation and release. In an in vitro study using MC3T3-E1 preosteoblast cells, increased expression of bone formation markers such as alkaline phosphatase (ALP), osteopontin (OPN), and osteocalcin (OCN) correlated with increased SrCO_3_ content in the capsules. The fabricated (Sr, Ca)­CO_3_ capsules are promising for bone tissue engineering applications, potentially improving bone regeneration by combining controlled Sr^2+^ release with drug release capabilities.

## Supplementary Material



## Notes

Results of elemental analysis by SEM–EDX of (Ca, Sr)­CO_3_ capsules under each preparation condition (PDF)

## †

Faculty of Pharmacy and Pharmaceutical Sciences, Josai University, 1–1 Keyakidai, Sakado, Saitama 350–0295, Japan
