# Supplementary material for: Preparation of Organic–Inorganic Hybrid (Sr, Ca)CO3 Capsules Based on Thermoresponsive Degradable Coacervation
Source: ACS Appl Bio Mater. 2025 Jul 24;8(8):7261–9. doi: 10.1021/acsabm.5c00954 (PMC12365917; doi:10.1021/acsabm.5c00954)
Supplement: Supplementary file 1 [file mt5c00954_si_001.pdf]

Supporting Information

Preparation of organic-inorganic hybrid (Sr, Ca)CO_3 capsules based on thermoresponsive degradable coacervation

Syuuhei Komatsu [1†*], Yuya Mizuno [1], Akihiko Kikuchi [1§*]

[1] Department of Materials Science and Technology, Tokyo University of Science

6-3-1 Niijuku, Katsushika, Tokyo 125-8585, Japan

† Current address: Faculty of Pharmacy and Pharmaceutical Sciences, Josai University, 1-1 Keyakidai, Sakado, Saitama 350-0295, Japan

§ Current address: Department of Medical and Robotic Engineering Design, Tokyo University of Science, 6-3-1 Niijuku, Katsushika, Tokyo 125-8585, Japan

* Corresponding author: Syuuhei Komatsu; Phone: +81-49-271-7943; FAX: +81-49-271-7567; E-mail: skomatsu@josai.ac.jp

Akihiko Kikuchi; Phone: +81-3-5876-1415; Fax: +81-3-5876-1639; E-mail: kikuchia@rs.tus.ac.jp

Contents

1. S1. Results of elemental analysis by SEM-EDX of (Ca, Sr)CO3 capsules under each preparation condition

Sr-3

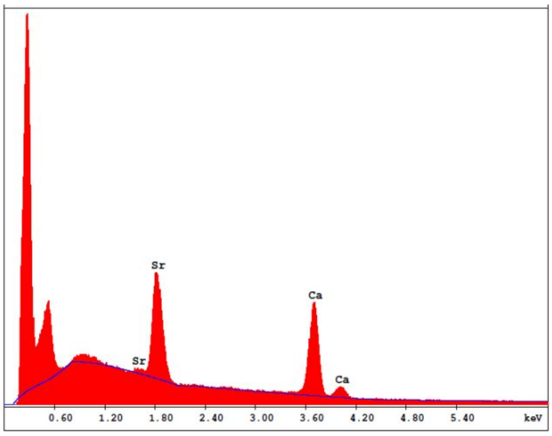

EDAX ZAF Quantification (Standardless)
Oxides
SEC Table : Default

Element	Wt %	Mol %	K-Ratio	Z	A	F
SrO	55.87	40.66	0.4041	0.8874	0.9606	1.0033
CaO	44.13	59.34	0.2751	1.0589	0.8236	1.0000
Total	100.00	100.00				

Element	Net Inte.	Bkgd Inte.	Inte. Error	P/B
SrL	108.58	31.35	0.54	3.46
CaK	105.48	12.46	0.48	8.46

Sr-5

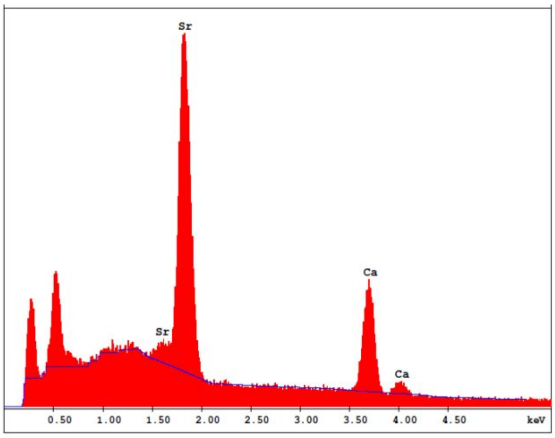

EDAX ZAF Quantification (Standardless)
Oxides
SEC Table : Default

Element	Wt %	Mol %	K-Ratio	Z	A	F
SrO	75.98	63.12	0.5755	0.9153	0.9771	1.0016
CaO	24.02	36.88	0.1446	1.0942	0.7695	1.0000
Total	100.00	100.00				

Element	Net Inte.	Bkgd Inte.	Inte. Error	P/B
SrL	434.10	53.64	0.76	8.09
CaK	155.60	26.58	1.31	5.85

Sr-7

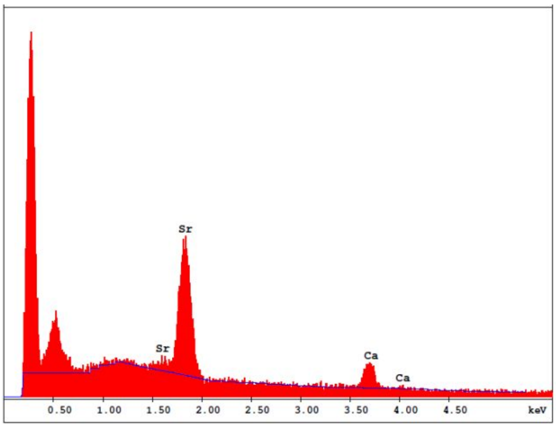

EDAX ZAF Quantification (Standardless)
Oxides
SEC Table : Default

Element	Wt %	Mol %	K-Ratio	Z	A	F
SrO	83.87	73.78	0.6472	0.9270	0.9833	1.0010
CaO	16.13	26.22	0.0958	1.1092	0.7494	1.0000
Total	100.00	100.00				

Element	Net Inte.	Bkgd Inte.	Inte. Error	P/B
SrL	114.36	21.94	1.56	5.21
CaK	24.16	10.38	3.92	2.33

Sr-10

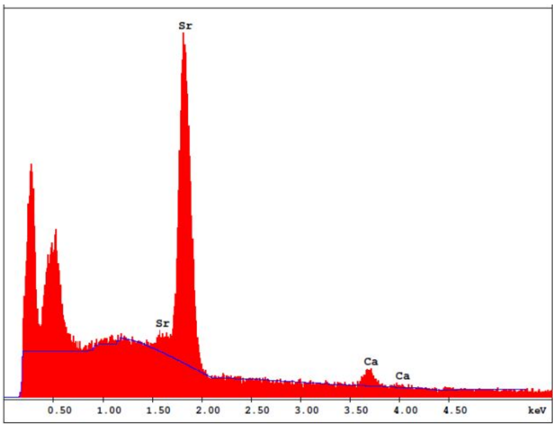

EDAX ZAF Quantification (Standardless)
Oxides
SEC Table : Default

Element	Wt %	Mol %	K-Ratio	Z	A	F
SrO	95.22	91.52	0.7550	0.9449	0.9920	1.0003
CaO	4.78	8.48	0.0279	1.1318	0.7216	1.0000
Total	100.00	100.00				

Element	Net Inte.	Bkgd Inte.	Inte. Error	P/B
SrL	334.00	42.02	0.87	7.95
CaK	17.60	16.18	5.68	1.09

Figure S1 Results of elemental analysis by SEM-EDX of (Ca, Sr)CO_3 capsules under each preparation condition
